# Supplementary material for: Black African men’s perception of mental health in Africa: Scoping review protocol
Source: PLOS Ment Health. 2026 Apr 3;3(4):e0000586. doi: 10.1371/journal.pmen.0000586 (PMC13048480; doi:10.1371/journal.pmen.0000586)
Supplement: Attachment — Submitted filename: Plos one point by point response final.pdf [file pmen.0000586.s002.pdf]

Dear PLOS Mental Health Journal editors

We have revised the protocol as per the reviewers' comments and suggestions. Below are the comments made by reviewers and our responses to the comments.

Point-by-point response letter to reviewers' comments

Reviewer 1 comments and authors' responses

Comment 1: The protocol does not clearly discuss how "African masculinity" is understood in different sub-regions. This could lead to inaccurate generalizations.

Authors' response: We agree, we have attended literature that indicates how masculinity is perceived in different African regions in the introduction.

Comment 2: The criteria for "Black African men" need to be clearer. It should be specified whether this includes individuals in the diaspora or only those living in Africa.

Authors' response: The criteria have been revised, and adult males residing within African countries have been added.

Comment 3. The plan for thematic analysis is not specific. The authors should indicate whether they will use inductive or deductive coding.

Authors' response: We agree; details on how thematic analysis will be done have been added.

Comment 4. It is important to state clearly whether the review includes studies of Black African men in Western countries or is only about those on the African continent, as the title suggests.

Authors' response: We agree on the eligibility criteria and study selection. We have already indicated within Table 1: Framework for determining the eligibility of the research question under the context section that only studies conducted within the African continent will be considered

Comment 5. When discussing mental health intervention strategies. It is suggested to refer from the paper: “A Systematic Review of Mental Health Monitoring and Intervention Using Unsupervised Deep Learning on EEG Data “. Including this suggested reference adds a modern view on mental health monitoring and intervention techniques. It strengthens the manuscript by showing advances in data-driven mental health practices, which contrast with the current focus on perceptions and qualitative methods.

Authors' response: We agree and appreciate the suggestion, and we will incorporate the suggestion when discussing the findings of the actual scoping review. So far, we feel that we do not have much data to base on in discussion on mental health intervention strategies.

Comment 6:The authors should explain what the "five-stage framework" is and how they will validate the themes, for example, through a third reviewer or software such as NVivo.

Authors' response: We agree, we have provided a brief description of the framework. We have also added measures that will be taken to ensure the validity of the results.

Comment 7: The term "Black African men" should be used consistently throughout the document, avoiding variations like "African men" or "men in the African context."

Authors' response: We agree, consistency on the use of the term “Black African men” has been ensured throughout the document.

Comment 8: While the protocol cites important works, it should also include recent systematic reviews on African mental health to provide a stronger foundation for the study.

Authors' response: We agree, we have added the findings of a systematic review conducted by Babajani et al(2024), which indicates that suicide attempt prevalence among African men was at 7.6%. This indicates how mental health is problematic among African men.

Reviewer 2 comments and authors response

Comment 1: Great topic with an excellent goal of improving policies on mental health for black African men. However, manuscript was presented as a research article but lacks the depth of information to substantiate the claim of a research article. It also needs more key words and improved grammatical sentences.

Authors' response: We agree and acknowledge that the paper is not a research article but a protocol

Comment 2: Since it's in a scoping review protocol phase, perhaps it would be better to present the research article for publication review after the final stage of analysis, results and synthesis (April-May). This will give the authors the chance to perform more in-depth analysis which also improves the depth of information for meeting the study's goal/purpose
Thank you for the invitation to review this manuscript.

Authors' response: We agree, the research article will be submitted for publication once the review is completed.

Reviewer 3 comments and the author's response

Comment 1:
Study Design:

Mention the steps of the scoping review, the one you followed in this paper Arksey O'Maley and give Headlines accordingly.

Authors' response: We agreed, the steps of scoping review according to Arksey O'Maley have been added on the study design section.

Comment 2

Inclusion and Exclusion Criteria:

We do not include the points in exclusion criteria which are already mentioned in the inclusion criteria. For example, the review includes english language literatures. You do not need to mention it in the exlusion criteria. Instead add points that are not in the inclusion criteria. You need to change the exclusion criteria completely. For instance, you can add- Articles related to mental health for women/girls in the study. I am giving you just examples to make it easier for you to understand.

Authors' response: We agree, we have revised the exclusion criteria

Author's additional comments

We have also revised the study topic by replacing the African continent with Africa.

Search structures have also been added in the search strategy and indicate the search queries being used to search data. There are also additional terms added on the search keywords and strategy.
